# The infection and impact of *Azorhizobium caulinodans* ORS571 on wheat (*Triticum aestivum* L.)

**DOI:** 10.1371/journal.pone.0187947

**Published:** 2017-11-30

**Authors:** Huawei Liu, Xiaojing Wang, Huaiting Qi, Qian Wang, Yongchao Chen, Qiang Li, Yuying Zhang, Li Qiu, Julia Elise Fontana, Baohong Zhang, Weiling Wang, Yingge Xie

**Affiliations:** 1 College of Life Sciences, Northwest A&F University, Yangling, Shaanxi, China; 2 College of Science, Northwest A&F University, Yangling, Shaanxi, China; 3 College of Veterinary Medicine, Northwest A&F University, Yangling, Shaanxi, China; 4 Department of Biology, East Carolina University, Greenville, NC, United States of America; Dokuz Eylul Universitesi, TURKEY

## Abstract

Based on our previous study, cereal crop wheat (*Triticum aestivum* L.) could be infected by rhizobia *Azorhizobium caulinodans* ORS571, and form para-nodules with the induction of 2.4-dichlorophenoxyacetic acid, a common plant growth regulator. To enhance this infection and the potential agricultural application, we compared six different infection methods (Direct seed dip; Seed germination dip; Pruned-root dip; Foliar spray; Circum-soil dip; Seed dip and circum-soil dip) for achieving the high efficient infection of *A*. *caulinodans* into wheat plants by employing a *green fluorescent protein (gfp)*-labeled *Azorhizobium caulinodans* strain ORS571. With proper methods, copious rhizobia could enter the interior and promote the growth of wheat to the hilt. Circum-soil dip was proved to be the most efficient method, seed germination dip and pruned-root dip is the last recommended to infect wheat, seed germination dip and seed dip and circum-soil dip showed better effects on plant growth, pruned-root dip did not show too much effect on plant growth. This study laid the foundation for understanding the interaction between rhizobia and cereal crops and the growth-promoting function of rhizobia.

## Introduction

It has been shown that associate nitrogen fixing bacteria (ANFB) are essential to plant growth promoting rhizobacteria (PGPR), which are of great importance to plants for promoting growth; the colonization site also play a key role [1–3]. ANFB belongs to PGPR, which could provide nitrogen nutrition to plants. Rhizobium *A*. *caulinodans* could act as a special kind of ANFB, which could colony inside plant para-nodules and show certain nitrogenase activity. ANFB is vital to boosting seedling vigor [4], strengthening plants’ utilization efficiency on nitrogen [5, 6] and absorption of minerals [7], facilitating absorbency of soluble phosphor salts [8], accelerating photosynthetic rate [9], enhancing roots respiration through generating phytohormones like IAA and GA[10, 11] and enlarging roots topology and available absorption area[10, 11]. There are great interests in establishing closed relationships between non-legumes and the nitrogen-fixing rhizobia. *Azorhizobium caulinodans* ORS571 (*A*. *caulinodans* ORS571) induce nodulation and nitrogen fixing of roots and stems of *Sesbania rostrata* (*S*. *rostrata*) through crevices [12]. Considering the looser demanding of oxygen than other rhizobia, *A*. *caulinodans* has become a vital growth promoting ANFB for graminaceous crops [13].

Rhizobia, the root-nodule endosymbionts of leguminous plant, also form natural endophytic associations with roots of cereal plants [14]. Rice inoculated with certain test strains of *gfp*-labeled rhizobia, such as *A*. *caulinodans* ORS571 and *Sinorhizobium meliloti* USDA 1002, improved significantly higher root and leaf biomass. It was also increased the photosynthetic rate, stomatal conductance, transpiration velocity, water utilization efficiency, flag leaf area in the infected rice plants; the infected plants also accumulated higher levels of indoleacetic acid and gibberellin growth regulating phytohormones. The endophytic bacteria were disseminated in both below-ground and above-ground tissues and enhancement of growth physiology. The results heightened its interest and potential value as a biofertilizer strategy for sustainable agriculture to produce the world’s most important cereal crops[15].

It was reported that para-nodules (nodular structures), induced by 2, 4-dichlorophenoxyacetic acid (2, 4-D) [16], in wheat roots could be infected by rhizobia like *A*. *Caulinodans* [17]. 2, 4-D targets wheat lateral root primordia which were then transformed to nodules, meanwhile, rhizobia invading these nodules via interspaces of epidermal cells surfaces, the way that resemble to the direct infection in legumes. *Gfp*—labeled rhizobia could colonize in the conjunction part of lateral roots between epidermal cells in rice, and that all isolated rhizobia were able to renodulate in their host plants. Rhizobia infected could be transferred upward into stem and leaves’ cells through inner lateral root cells[18].

Within the same condition, the dinitrogenases activity of *A*. *caulinodans* was only one-eight in wheat compared with which was shown in their natural host sebania, the dry weight and total nitrogen of infected wheat was significantly increased compared with control [19]. Wheat with *A*. *caulinodans* has a higher speed on its roots growth generating a great amount of lateral roots. This research also indicated that *A*. *caulinodans* could improve wheat biomass and total nitrogen, accelerate plants growth[19]. After it was induced with low concentration 2, 4-D solution and forming nodule-structure tissue (nodule-like), which has been done by Nie[16], *A*. *caulinodans* could infect and perform reproduction in the crevice of cells and within them, providing their host wheat 16–23% nitrogen.

Wheat plants formed nodule-like tumors (para-nodules) when inoculated with rizobium and treated with synthetic plant growth regulator, 2, 4-D. Wheat seedlings treated with low concentrations (1.0 ug.g-1) of 2, 4-D developed nodule-like structures (para-nodules) mainly along primary roots. *A*. *caulinodans* colonized para-nodules externally at the junction of nodules and roots as well as at the top of nodules[20].

In this study, we compared 6 different infection methods that examining *A*. *caulinodans’* infection efficiency and the growth-promoting effects on wheat plants, to form the rudiment of studying the distribution pattern and the molecular mechanism of *A*. *caulinodans* infect wheat seedlings, to shed a light on applying *A*. *caulinodans* to agricultural production.

## Materials and methods

### Wheat, bacterial strain and culture

Seeds of wheat (*Triticum aestivum* L.) cv. Xiaoyan 22 and *gfp* -labeled *A*. *caulinodans* (*gfp*-*A*. *caulinodans*) were maintained by our laboratory. TY medium contained 5g·L^-1^tryptone, 3g·L^-1^ yeast powder, and 0.88g·L^-1^ CaCl_2_·2H_2_O with pH7.4. YMA solid medium contained 10g·L^-1^ mannitol, 3g·L^-1^ yeast powder, 0.5g·L^-1^ K_2_HPO_4_, 0.2g·L^-1^ MgSO_4_·7H_2_O, 18g·L^-1^ agar powder with pH7.0; both were used for verifying whether the disinfection was successful. Rhizobia culture condition: 28°C, 220 rpm to an exponential growth period (about 1.0×10^9^ per milliliter). Wheat plants were cultured as following conditions: 16 h light with 25°C temperature, relative humidity was 46%; darkness duration: 8h with 18°C temperature and 37% relative humidity.

### Wheat seed pretreatment and culture

Wheat seeds were first sterilized with 75% alcohol for 30 seconds and then washed three times with sterile water following by immersed in 1% sodium hypochlorite for 10 minutes and washed three times using sterile water. Ten of these sterilized seeds were put on a plate of YMA solid medium that were then cultured at dark and 28°C to check whether the sterilization was successful. Sterilized wheat seeds were placed on sterilized petri dishes that were covered with two aseptic wet filter papers to give these seeds a moist surrounding to germinate in darkness for 36 hours.

Perlite-Vermiculite (1:2) were used as culture media for wheat seedling growth. Wheat seedling culture condition: light duration16h, temperature 25°C, relative humidity 46%; darkness duration 8h, temperature 18°C, relative humidity 37%. Nourished with 1/2 Hoagland’s nutrient solution 50 mL every 3 days. Each experiment had 5 biological replicas, each of them contained 6 germinated seedlings.

### Suspension culture of *gfp*-*A*. *caulinodans*

A single *gfp*-*A*. *caulinodans* bacterial colony was cultured in TY medium with tetracycline and ampicillin. *A*. *caulinodans* was cultured under following conditions: 28°C, 220 rpm to an exponential growth period. Then, *gfp*-*A*. *caulinodans* were collected in 50 milliliter tubes after centrifuging 20 minutes with 8000 rpm. Precipitated bacteria were diluted to 1×10^9^ per milliliter with PBS solution, and 1 ug.g^-1^ 2, 4-D were added into the bacteria.

### gfp-*A*. *caulinodans* infection

Six different methods were employed to infect wheat. Out of these methods, seed germination dip (GD) and seed dip and circum-soil dip (SD & FS) methods were developed my ourselves, and the rest were followed a previous report [21].

Direct seed dip [22]: surfaces’ sterilized seeds were soaked in bacterial solution diluted by PBS buffer for 2 hours as treatment; at the same time, the seeds treated with aseptic water were served as controls.

Seed germination dip (GD): wheat seeds were firstly germinated and then the germinated seeds were soaked in 1 milliliter of diluted ORS 571 PBS solution for 2 minutes.

Pruned-root dip (PD): both main and lateral roots were clipped 50% of the total length using aseptic scissors and tweezers, then the pruned roots were soaked in 1 milliliter of diluted bacterial PBS solution.

Foliar spray (FS): aseptic swab dipped with 1 milliliter of diluted *A*. *caulinodans* PBS solution were sprayed onto leaves (both sides).

Circum-soil dip (CD): 1 milliliter of diluted *A*. *caulinodans* PBS solution were dipped onto peripheral soil while germinated seeds were transplanted into soil instead of petri dish. Dipped solution was not allowed to contact directly with seeds.

Seed dip and circum-soil dip (SD & FS): combine SD and FS together for bacterial infection.

### Observation of bacterial infection

Fluorescence microscopy was employed to monitor the green fluorescence in wheat tissues, which was generated by gfp- *A*. *caulinodans*. Root and leaf tissues were cut into small slice sections which were inspected by fluorescence microscopy scanning up-to-down layers to observe the distribution of inner gfp- *A*. *caulinodans*. The green fluorescence was compared among different infection methods.

### Effects of different infection methods on plant growth and development

After 7 and 14 days of *A*. *caulinodans* infection, wheat seedlings were measured for different traits independently. The effect of different infection method on wheat plant development was observed, which included root length, leaf length, root number, leaf number, and shoot dry weight. Each treatment and control groups contained 5 biological replicates and each biological replicate contained 5 seedlings.

## Results

### Infection of *A*. *caulinodans* and its migration in wheat plants

**Fig 1:**
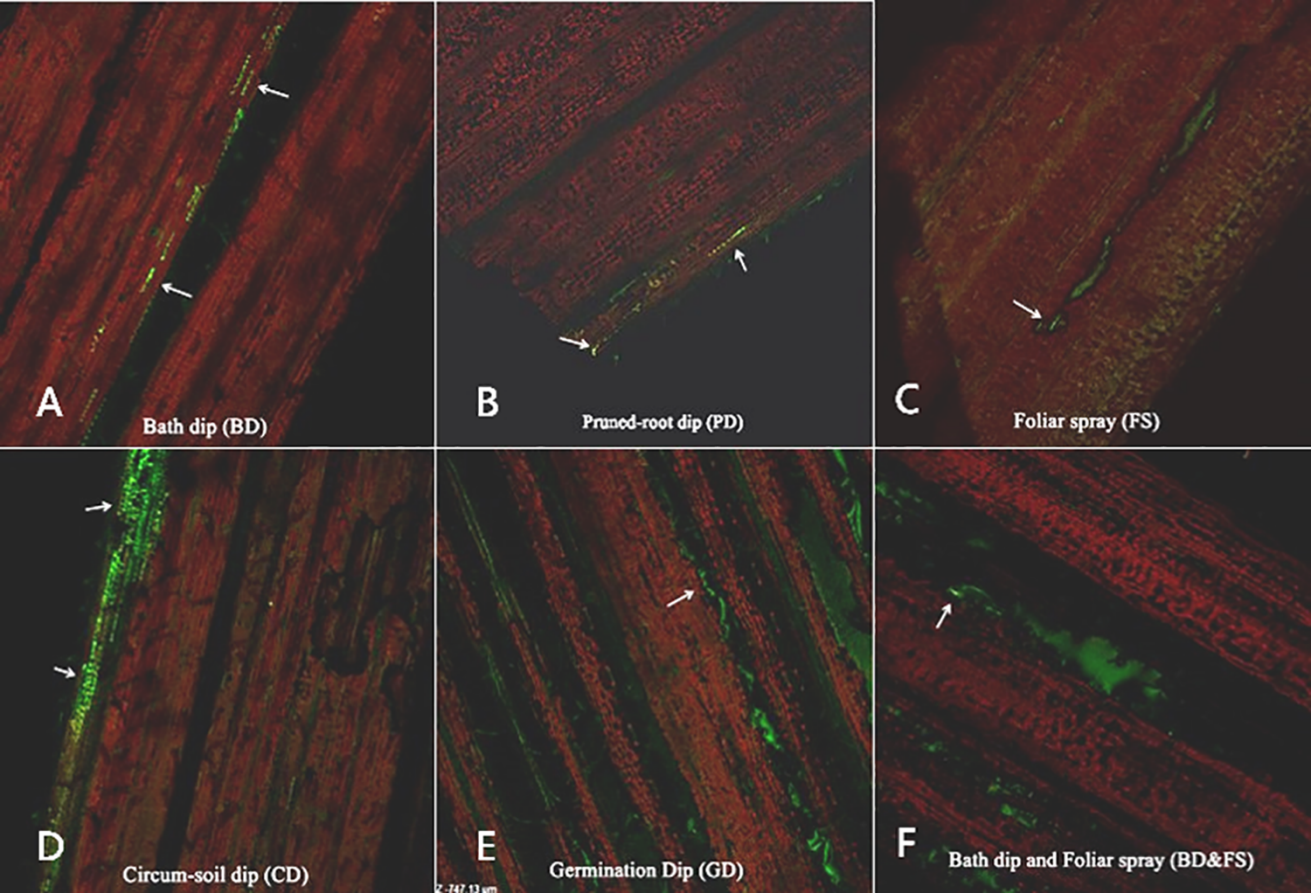
The distribution of *gfp*-*A*. *caulinodans* ORS571 within leaves of all six infection methods. Arrows refer to appearance and position of *gfp*-*A*. *caulinodans*. (A) Seed Dip (SD). Infected rhizobia GFP signal was observed around the middle part of vein; (B) Pruned-root Dip (PD). A certain number of rhizobia was distributed along the leaf margins; (C) Circum-soil Dip (CD). A great number of detectable rhizobia were along the leaf blades. (D) Foliar Spray (FS). Rhizobia infected a relatively wide area in leaf blade. (E) Seed Germination Dip (GD). A spot of rhizobia was around the vein. (F) Seed dip and circum-soil dip (SD & FS). Given mass of rhizobia both around vein and leaf margin. Considering all these six images, as to wheat leaves, CD is the best way for rhizobia to infect, followed by SD&FS. SD, FS, PD generated less infection. The last recommended method is GD.

All six tested methods can be used to infect *gfp*-*A*. *caulinodans* to wheat leaves (Fig 1). After *gfp*-*A*. *caulinodans* infection, *gfp*-*A*. *caulinodans* can be found in wheat leaves in all treated groups. In seed dip (SD) treatment, infected rhizobia GFP signal was observed around the middle part of vein. In pruned-root dip (PD) treatment, a certain number of rhizobia were found along the leaf margins. In circum-soil dip (CD) treatment, a great number of rhizobia were observed along the leaf blades. In foliar spray (FS), Rhizobia infected a relatively wide area in leaf blade. In seed germination dip (GD) treatment, a spot of rhizobia was found in the vein. In seed dip and circum-soil dip (SD & FS) treatment, many rhizobia were existed in both vein and around leaf margin. Compared one method with another, as to wheat leaves, CD is the best way for rhizobia to infect, secondly is SD&FS, followed by SD, FS, and PD. GD seems is less efficient for infection of *gfp*-*A*. *caulinodans*.

**Fig 2:**
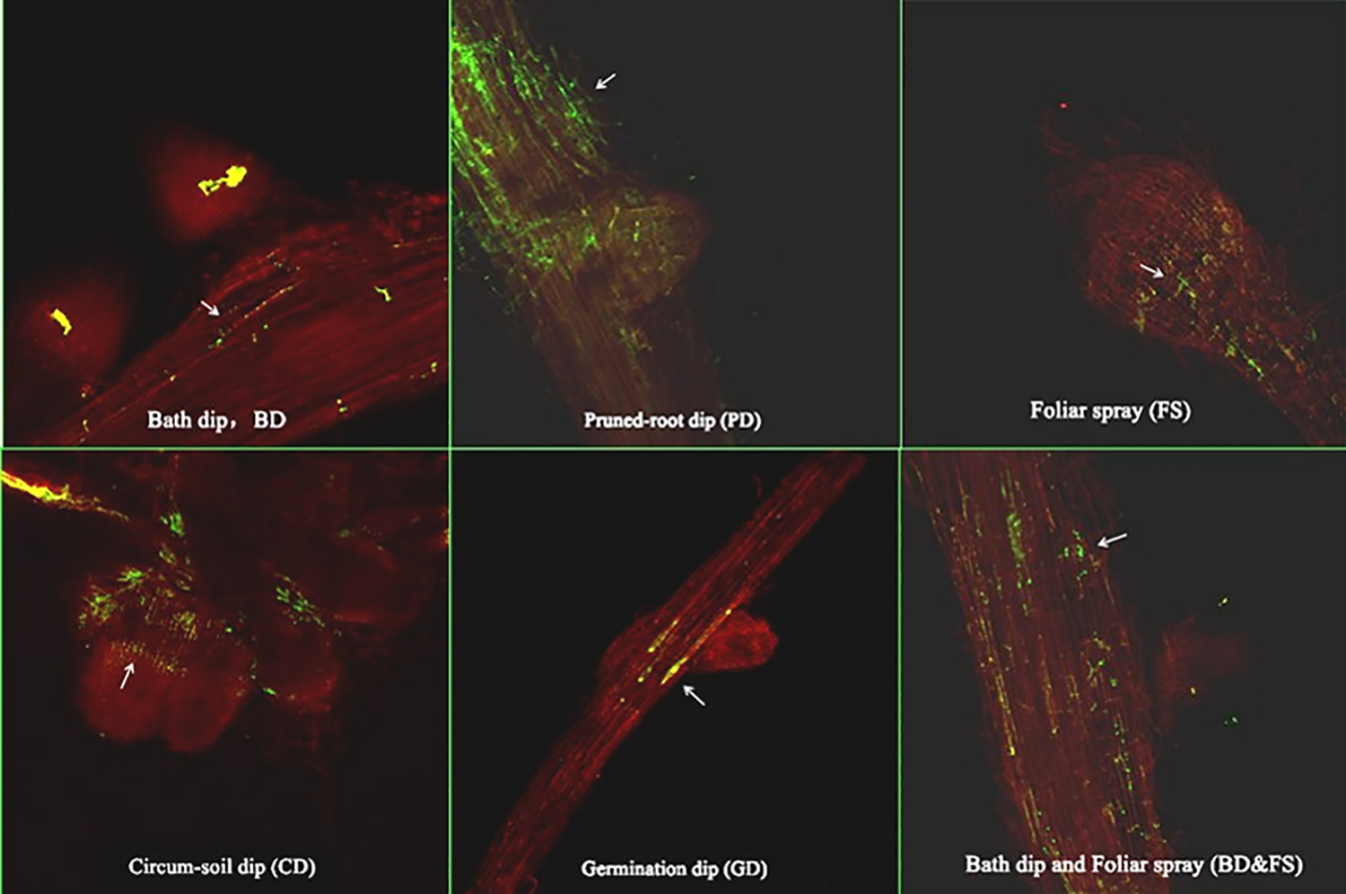
The distribution of *gfp*-*A*.*caulinodans* in root tissue. In order to make samples easy, we added 2, 4-D as an inducer to all methods to form para-nodules, a specific tissue that can be used as perfect target to trace rhizobia with GFP fluorescence. Arrows refer to the appearance and position of rhizobia ORS 571. (A) SD, a few of rhizobia existed within para-nodules. (B) PD, a certain amount of rhizobia is in para-nodules and root cortex. (C) CD, a relatively high amount of rhizobia can be detected in para-nodules. (D) FS, relatively low amount of rhizobia was in para-nodules. (E) GD, we can hardly saw any rhizobium in para-nodule of this method. (F) SD&FS, there were rhizobia within para-nodules primordia, but no one within inside. Comparing all these methods, CD is still the best way for roots, secondly is SD&FS, thirdly FS, PD, SD, GD has no support to be a good way to infect wheat.

All six tested methods can also be used to infect *gfp*-*A*. *caulinodans* to wheat root tissues (Fig 2). After *gfp*-*A*. *caulinodans* infection, *gfp*-*A*. *caulinodans* can be found in/on wheat roots in all treated groups. During SD treatment, few rhizobia were observed within para-nodules. During PD treatment, a certain amount of rhizobia were found in both para-nodules and root cortex. In CD treatment, a relatively high amount of rhizobia were observed in the para-nodules. In FS treatment, relatively low amount of rhizobia was observed in the para-nodules. In GD treatment, it was difficult for *gfp*-*A*. *caulinodans* infecting wheat roots and the para-nodule. In SD&FS treatment, there were rhizobia within para-nodules primordia, but no one within inside. Comparing all these methods, as observation in wheat leaves, CD is the best method for *gfp*-*A*. *caulinodans* infection, secondly is SD&FS, followed by FS, PD, and SD. However, GD was not an efficient method for *gfp*-*A*. *caulinodans* infection.

### Effect of *A*. *caulinodans* infection on wheat plant growth and development

**Fig 3:**
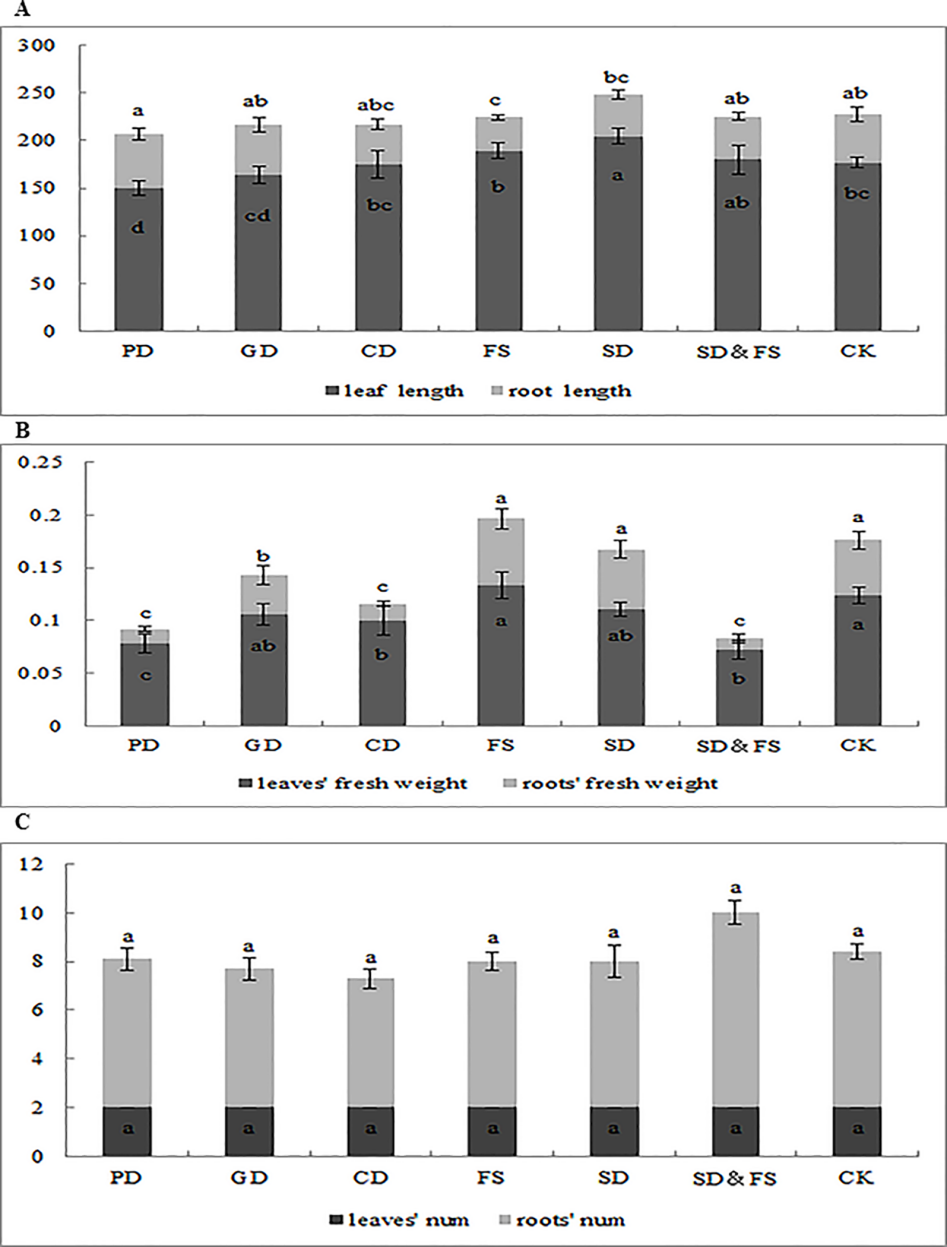
The effect of different infection methods on wheat plant growth and development. (A) the length of leaves and roots, only SD and SD&FS are better than CK; (B) weight of leaves and roots after infection. FS is better than CK, whereas PD is worse than CK; (C) the number of leaves and roots. Only CD is worse than others. The data represents the means of 3 biological replicates, error bars represent SEM.

*A*. *caulinodans* infection affected wheat plant growth and development, evidenced by the number of leave and roots as well as their weight (Fig 3). SD and SD&FS infections strengthened wheat leaf and root growth. FS infection increased plant biomass.

## Discussion

In this study, we compared six different infection methods, our results show that the different infection methods varied on the aspect of infection efficiency and its impact on plant growth, and the infection efficiency and growth-promoting were tightly associated with the methods we used. All tested methods did not do substantial harm to wheat; CD, SD & FS and FS were most efficient ways to allow *A*. *caulinodans* infect wheat plants.

CD is the best way to infect wheat plants. Rhizobia can be clearly detected in both leaves and para-nodules induced by 2, 4-D, and the number of *A*. *caulinodans* cells was relatively high (Figs 1C and 2C). However, this method may cause negative impacts on plant growth, evidenced by the length of leaves and roots (Fig 3A) and the total fresh weight and the number of roots (Fig 3B). This probably can be explained by the mass quantity of live rhizobia. When such a great number of rhizobia were added, they competitively seized nutrients from wheat plants affecting the regular growth of wheat roots and indirectly affected plant growth and finally the plant biomess.

SD & FS treatments also allowed rhizobia *gfp*-*A*. *caulinodans* to infect both wheat leaves and roots. The difference from other methods was that the distribution of the GFP signal was in the middle part of leaf, instead of leaf margin, and that the signals in the roots were mainly in the inner root tissue, rather than the para-nodules or primordia (Figs 1F and 2F). After the treatment, *A*. *caulinodans* enhanced plant growth compared with the untreated controls but lower than the plants treated by SD method (Fig 3A). Compared to SD and FS methods, more number of *A*. *caulinodans* infected the plants, which may compete the nutrients with the plants and caused a little bit of lower plant biomass than that of SD or FS treated plants, independently.

Significant amount of fluorescence was observed on the margin area of wheat leaves after rhizobia *gfp*-*A*. *caulinodans* infection using FS treatment. However, the fluorescence signals were lower than that caused by CD and SD treatments, and the infected location is different from that caused by CD treatment (Fig 1D) in which fluorescence was detected within the para-nodules, not in the primordia (Fig 2D). Once the bacteria entered into the plant tissue, endophytic bacteria either remained in a specific plant tissue like the root cortex or colonized the plant systematically by transporting through the conducting elements or apoplast. Microscopy studies demonstrated that spread of bacteria onto undamaged leaf surfaces, or inoculated into guttation droplets from hydathodes, were present in leaf intercellular spaces and in xylem vessels. FS treatment enhanced wheat plant growth and development, the fresh weight was the highest one in all six tested methods (Fig 3A and 3B). This was mainly contributed by the rhizobia on surface of leaves.

Using SD infection method, less rhizobia fluorescence was detected in the middle of leaves than that using CD infection method. Although GFP was observed in the roots but only in mature para-nodules (Figs 1A and 2A). It was evident that *gfp*-*A*. *caulinodans* introduced as SD colonized the internal tissues of root radicles as they emerged from the seed coat; the similar phenomena was also observed by Mahaffe [23]. SD infection was the best method for enhancing plant root development. One of the potential reasons is that the rhizobia ORS571, attaching on the surface of roots, played a promoting role in the inner of roots as they entered. SD infection method also enhance plant growth evidenced by the fresh weight that was higher than other infection methods.

After *gfp*-*A*. *caulinodans* infection using PD method, less GFP fluorescence signals were observed in the margin area of leaves (Fig 1B). Roots’ fluorescence was relatively higher and distributed largely on primordia (Fig 2B). Besides providing entry avenues, wounds also created favorable conditions for the approaching bacteria by allowing leakage of plant exudates, which served as a food source for the bacteria. Because of breaks in the endodermis at these points, bacteria colonized in the cortex can cross the endodermis into vascular tissue and stem tissue [21]. We also saw GFP fluorescence in xylem of intact roots; the same results were also observed by Philip [11]. The length of roots was behind the other methods. This may due to the damages in roots at their early age. Similarly, the fresh weight was also the lowest in all, but the number of leaves and roots were not affected (Fig 3A and 3B).

GD treatment generated less rhizobia ORS571 in plants, in which the plant produced the similar signals as that in PD treatment and the affected location is similar as SD treatment (Fig 1E). We didn’t see any GFP fluorescence in roots, except the autofluorescence in roots and para-nodules (Fig 2E). In physiological indicators, length was corresponded with other methods and weights was in average status, roots number slightly bellowed than the controls and other treated groups (Fig 3A, 3B and 3C). This may be caused by the fact that the seedlings were too fragile to absorb a great number of rhizobia.

Overall, the infection efficiency varied with the methods used. On average, CD, SD and FS were most efficient ways to obtain maximal rhizobia.

## References

[1] Goswami D, Thakker JN, Dhandhukia PC. Portraying mechanics of plant growth promoting rhizobacteria (PGPR): A review. Cogent Food & Agriculture. 2016;2(1):1127500.

[2] Grobelak A, Napora A, Kacprzak M. Using plant growth-promoting rhizobacteria (PGPR) to improve plant growth. Ecological Engineering. 2015;84:22–8.

[3] Gupta G, Parihar SS, Ahirwar NK, Snehi SK, Singh V. Plant growth promoting rhizobacteria (PGPR): current and future prospects for development of sustainable agriculture. J Microb Biochem Technol. 2015;7(2):096–102.

[4] Biswas JC, Ladha JK, Dazzo FB, Yanni YG, Rolfe BG. Rhizobial inoculation influences seedling vigor and yield of rice. Agronomy Journal. 2000;92(5):880–6.

[5] Rasmussen M. Prokaryotic Nitrogen Fixation: a Model System for the Analysis of a Biological Process. ASM News. 2003:737–60.

[6] Yang J, Li H, Zhang D, Wu M, Pan B. Limited role of biochars in nitrogen fixation through nitrate adsorption. Science of The Total Environment. 2017;592:758–65. doi: 10.1016/j.scitotenv.2016.10.182 28341466 10.1016/j.scitotenv.2016.10.182

[7] Biswas JC, Ladha JK, Dazzo FB. Rhizobia inoculation improves nutrient uptake and growth of lowland rice 2000;64:1644–50.

[8] Yanni YG, Rizk RY, El-Fattah FKA, Squartini A, Corich V, Giacomini A, et al The beneficial plant growth-promoting association of Rhizobium leguminosarum bv. trifolii with rice roots. Functional Plant Biology. 2001;28(9):845–70.

[9] Peng S, Biswas JC, Ladha JK, Gyaneshwar P, Chen Y. Influence of rhizobial inoculation on photosynthesis and grain yield of rice. Agronomy Journal. 2002;94(4):925–9.

[10] Phillips DA, Joseph CM, Yang G-P, Martínez-Romero E, Sanborn JR, Volpin H. Identification of lumichrome as a Sinorhizobium enhancer of alfalfa root respiration and shoot growth. Proceedings of the National Academy of Sciences. 1999;96(22):12275–80. 10.1073/pnas.96.22.12275 PMC22907 10535912

[11] Phillips DA, Martinez-Romero E, Yang G-P, Joseph CM. Release of nitrogen: a key trait in selecting bacterial endophytes for agronomically useful nitrogen fixation The quest for nitrogen fixation in rice International Rice Research Institute, Manila, The Philippines 2000:205–17.

[12] Ndoye I, DeBilly F, Vasse J, Dreyfus B, Truchet G. Root nodulation of Sesbania Rostrata. Journal of Bacteriology. 1994;176:1060–8. 8106317 10.1128/jb.176.4.1060-1068.1994 PMC205157

[13] Stone PJ, O'Callaghan KJ, Davey MR, Cocking EC. Azorhizobium caulinodans ORS571 colonizes the xylem of Arabidopsis thaliana. Molecular plant-microbe interactions. 2001;14(1):93–7. doi: 10.1094/MPMI.2001.14.1.93 11194878 10.1094/MPMI.2001.14.1.93

[14] Yanni YG, Rizk RY, Corich V, Squartini A, Ninke K, Philip-Hollingsworth S, et al Natural endophytic association between Rhizobium leguminosarum bv. trifolii and rice roots and assessment of its potential to promote rice growth. Plant and Soil. 1997;194(1–2):99–114.

[15] Chi F, Shen S-H, Cheng H-P, Jing Y-X, Yanni YG, Dazzo FB. Ascending migration of endophytic rhizobia, from roots to leaves, inside rice plants and assessment of benefits to rice growth physiology. Applied and environmental microbiology. 2005;71(11):7271–8. doi: 10.1128/AEM.71.11.7271-7278.2005 16269768 10.1128/AEM.71.11.7271-7278.2005 PMC1287620

[16] Nie YF. Researches about inducing para-nodules in no-nodule plant. Nature. 1983;6:326–37.

[17] Kennedy IR, Pereg-Gerk LL, Wood C, Deaker R, Gilchrist K, Katupitiya S. Biological nitrogen fixation in non-leguminous field crops: facilitating the evolution of an effective association between Azospirillum and wheat Opportunities for Biological Nitrogen Fixation in Rice and Other Non-Legumes: Springer; 1997 p. 65–79.

[18] Ji K-X, Chi F, Yang M-F, Shen S-H, Jing Y-X, Dazzo FB, et al Movement of rhizobia inside tobacco and lifestyle alternation from endophytes to free-living rhizobia on leaves. J Microbiol Biotechnol. 2010;20(2):238–44. 20208425

[19] Sabry SR, Saleh SA, Batchelor CA, Jones J, Jotham J, Webster G, et al Endophytic establishment of Azorhizobium caulinodans in wheat. Proceedings of the Royal Society of London B: Biological Sciences. 1997;264(1380):341–6.

[20] El-Shahed AM, Abdel-Wahab MA. Para-nodule induction in wheat, maize and rice with 2, 4-D and its infection with Nostoc rivulare Kutzing. Pak J Biol Sci. 2006;9(9):1693–9.

[21] Bressan W, Borges MT. Delivery methods for introducing endophytic bacteria into maize. BioControl. 2004;49(3):315–22.

[22] Guddeti S, Zhang DC, Li AL, Leseberg CH, Kang H, Li XG, et al Molecular evolution of the rice miR395 gene family. Cell research. 2005;15(8):631–8. Epub 2005/08/25. doi: 10.1038/sj.cr.7290333 . 16117853 10.1038/sj.cr.7290333

[23] Mahaffee WF, Kloepper JW. Temporal changes in the bacterial communities of soil, rhizosphere, and endorhiza associated with field-grown cucumber (Cucumis sativus L.). Microbial Ecology. 1997;34(3):210–23. 9337416 10.1007/s002489900050

